# Standardized endotracheal tube and intravascular access placement in infants born at 22-23 weeks gestation

**DOI:** 10.1038/s41390-025-04186-8

**Published:** 2025-06-18

**Authors:** Nima Naseh, Linda Wallström, Richard Sindelar, Johan Ågren

**Affiliations:** Department of Women’s and Children’s Health, Uppsala University, Uppsala, Sweden

## Abstract

**Background:**

Recommendations are limited regarding the placement of oral endotracheal tube (ETT), and umbilical arterial/venous catheter (UAC/UAC) in the tiniest extremely preterm infants. We aimed to determine optimal insertion depths, and assess the impact of a too deep ETT position on outcomes.

**Methods:**

All infants born at 22-23 weeks gestation in 2019-2024 at Uppsala University Hospital, Sweden, were evaluated radiologically for accurate positions defined as: ETT (not right-sided/in main bronchus), UAC (T6-9 or L3–4), and UVC (right atrium/inferior vena cava junction). ETT position was further analyzed in relation to time to first extubation, respiratory severity score, duration of mechanical ventilation, bronchopulmonary dysplasia, and mortality.

**Results:**

The cohort (*n* = 75; 22w *n* = 39; 23w *n* = 36) had a survival rate of 41 and 64%, respectively. The ETT was accurately placed in 75%, and lower birth weight was associated with a too deep tip position (*p* = 0.018). The optimal median (IQR) insertion depths were: ETT 5.5 (5.5–6.0); low UAC 6.0 (5.5–6.5); high UAC 9.6 (9.2–10.3), and UVC 5.5 (5.0–6.1) cm. ETT position was not associated with respiratory outcomes or mortality.

**Conclusion:**

The suggested insertion depths can be expected to result in accurate positioning of ETTs and umbilical lines in infants born at 22-23 weeks gestation.

**Impact:**

- There is limited information to guide delivery room placement of endotracheal tube (ETT) and umbilical catheters (UC) in infants born at a gestational age (GA) of 22–23 weeks.
- An evaluation standardized insertion depths for ETT and UC, with use of x-ray based measurements of their positions, demonstrate the feasibility of using GA-based insertion depths.
- The suggested insertion depths can be expected to result in accurate ETT and UC tip positions in infants born at 22-23 weeks.

## Background

A majority of infants born at the lowest gestational ages (GA), i.e. those born at ≤23 weeks’ gestation, require endotracheal intubation for mechanical ventilation (MV) and/or surfactant administration, as well as intravascular access for blood sampling, invasive blood pressure monitoring, and parenteral nutrition. Accurate endotracheal tube (ETT) position is important since a too deep placement might lead to a less than optimal oxygenation/ventilation, and an uneven lung expansion carries an increased risk for atelectasis and/or air leak.^1–3^ In addition, malpositioning of umbilical arterial and venous catheters (UAC/UVC) has been associated with complications such as vessel perforation with pericardial/pleural effusions, hepatic necrosis, arrhythmias, and thrombosis.^4,5^

With increasing survival among infants born at 22-23 weeks gestation, the group referred to as “extremely preterm” i.e. born at less than 28 weeks, consists of infants with a 3-fold difference in birth weight, i.e. 400 to 1200 g.^6^ While this group’s heterogeneity is evident during their delivery room (DR) management, the evidence to guide the placement of ETT, and umbilical lines in infants born at 22-23 weeks, is limited.

In infants born at 22-23 weeks of gestation, the trachea has been measured to be 20–22 mm, from cricoid to carina.^7^ Given the movement of the infant during cares, this would leave approximately 10 mm at the mid-tracheal level (corresponding to the T1 vertebral body) that could be considered optimal for initial ETT placement.^7–10^ The most commonly applied formula for estimating ETT insertion depth in neonates is based on body weight, and although efforts have been made to adapt it for infants <1000 g, a too deep ETT placement still occurs frequently in these patients.^11–15^

The placement of umbilical lines in the tiniest infants is also uncertain with current recommendations offering little guidance.^1,16^ The most frequently used methods are based on surface anatomy measurements, such as the distance between the shoulder and umbilicus, and formulas based on birth weight, but has a poor success rate in terms of achieving a correct position.^17–20^

Based on the use of a standardized GA-based protocol, we aimed to evaluate the insertion depths of endotracheal tubes (ETT), and umbilical lines in infants born at 22–23 weeks of gestation. In addition, we aimed to investigate the association between a too deeply positioned ETT and respiratory outcomes, and mortality.

## Methods

### Study setting and management protocol

Retrospective single-center cohort study at Uppsala University Children’s Hospital, a regional referral tertiary care center providing active care to all infants born at 22-23 weeks’ gestation. Pregnant women with threatened labor are referred for obstetric care with the intention to prolong pregnancy and allow for antenatal steroid treatment, given as early as at 21 + 6 weeks.

If labor is imminent and prenatal transport is deemed unsafe, a transport team (neonatologist/neonatal nurse) is dispatched from the regional center to retrieve the infant from the delivery hospital.

According to national guidelines, cesarean section is not performed for fetal indication before 23 weeks.^21^

In brief, the institutional DR management protocol at 22-23 weeks’ gestation (Supplemental material) is as follows:

- Immediate DR intubation (oral route) with a size 2.0 F endotracheal tube (ETT) at a lip-tip distance of 5.5 cm.
- All respiratory support provided with a neonatal ventilator.
- Early instillation of 80 mg Surfactant (Poractant alfa) in the DR.
- Placement of umbilical catheters (arterial and venous) to a depth of 6 cm from the skin surface.

At NICU admission, chest and abdominal X-rays are taken to verify the positions of the ETT and the umbilical lines.

### Subjects

All inborn infants, or those admitted within 24 h of birth, with a gestational age (GA) of 22 + 0 to 23 + 6 weeks during the period of January 1, 2019 to July 1, 2024, were included.

### Demographics and outcome definitions

All data were collected from the electronic patient records, and the electronic patient data management system (MetaVisionNICU, iMDSoft, Tel Aviv, Israel), and included gestational age (GA) as estimated by routine first and/or second trimester ultrasound, antenatal steroid exposure and timing of administration, mode of delivery, birth weight, Apgar scores, and sex. The respiratory outcomes included the time to first extubation attempt, the respiratory severity score (RSS)^22^ at day 1 and 7, the total duration of and age at final weaning from MV, and the diagnosis of bronchopulmonary dysplasia (BPD) according to Jensen et al. ^23^ Outcomes were compared between infants with a too deeply positioned ETT vs the rest of the cohort. To assess the potential impact of asymmetric ventilation, the latter group also included infants with a too highly positioned ETT. Necrotizing enterocolitis (NEC) was diagnosed with histopathological diagnosis after laparotomy, or at autopsy. Intraventricular hemorrhage (IVH) was diagnosed by ultrasound and staged according to Papile.^24^ With the unit’s practice of conservative management of patent ductus arteriosus (PDA), treatment refers to those surgically ligated. Survival was to discharge home.

### Evaluation of ETT and UC positions

**Fig. 1:**
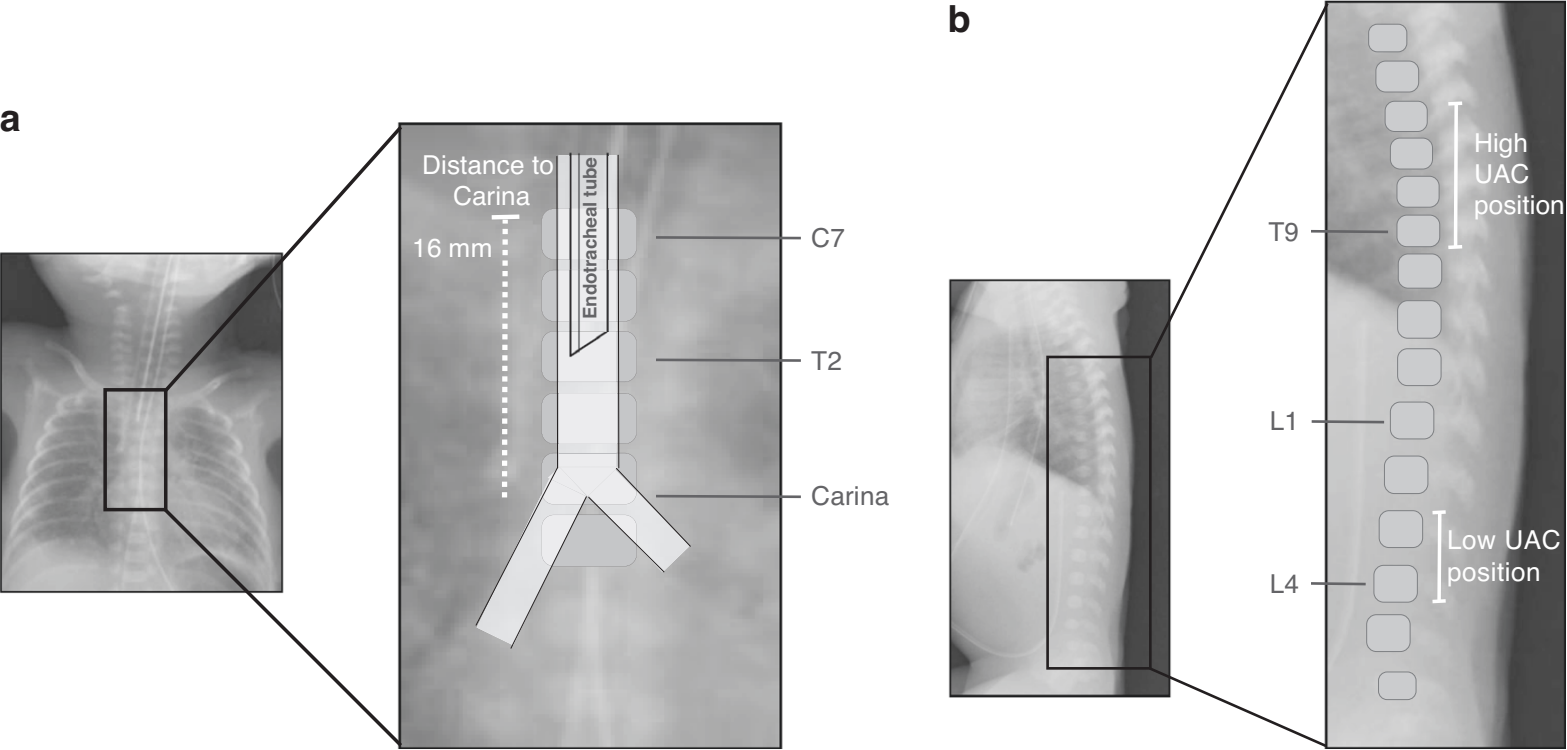
Representative chest x-rays with enlarged box to illustrate the radiological features. **a** Endotracheal tube position (C, Cervical; T, Thoracic). **b** Umbilical artery catheter (UAC) position (T, thoracic; L, Lumbar).
- UAC: Position based on the tip in relation to the vertebral bodies^4,26^ with a correct low position defined as the tip placed at L3–L4, and a correct high position at T6–T9 (Fig. 1b).
- UVC: Correct position defined as the UVC tip placed at the level of the diaphragm ± 5 mm^16,27^ (Fig. S1).

Insertion depths are routinely documented at insertion or at admission, using the measure from the lip (ETT), or skin plane (UCs). In addition to the initial routine assessment by a pediatric radiologist, chest and abdominal X-ray films following DR management were reviewed by a neonatologist (N.N), blinded to the individual infants’ clinical characteristics, outcomes, and the assessment by the pediatric radiologist. The review was performed using clinical-radiological software (Vue PACS, Carestream Health, Rochester, NY), and their initial positions were evaluated in relation to the following pre-defined criteria:

- ETT: Position primarily based on the measured tip-carina distance, and secondarily on its relation to vertebral bodies.^8,25^ A correct position was defined as the ETT not deviating to the right, or being in the main bronchus, or above the body of C7 (Fig. 1a). In addition, an optimal ETT tip position was also analyzed in relation to mid-tracheal, between T1 to T2.

### Statistical analyses

Data are presented as *n* (%), mean ± SD, or median (IQR). Group comparisons, based on GA (22 vs. 23 weeks) and ETT positioning (correct vs. incorrect), used Pearson’s χ2 or Fisher’s exact test for categorical variables, and Student’s *t*-test or Mann-Whitney U for continuous variables. Significant associations between ETT/UC and respiratory outcomes were further analyzed using multivariable and logistic regression models (SPSS v29, SPSS Inc., Chicago, IL). A *p* < 0.05 was considered statistically significant.

## Results

**Fig. 2:**
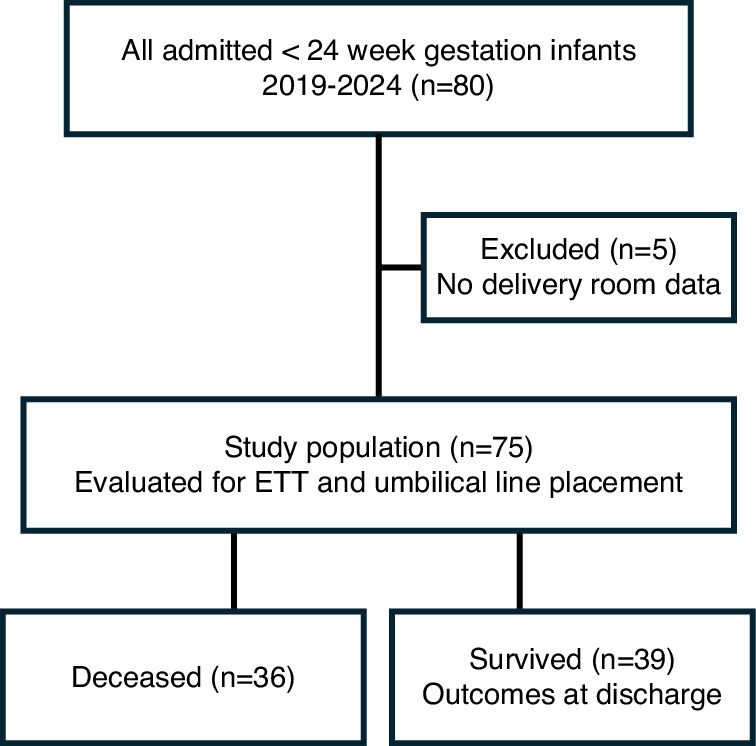
Flowchart of the study cohort. STROBE diagram showing included infants < 24 weeks gestation; Final cohort (*n* = 75) evaluated for ETT and UC placement and outcomes.

A total of 80 infants were born during the study period, all were intubated in the DR and survived to NICU admission. After exclusion of infants in whom initial ETT/UC data were missing (*n* = 5), the final cohort (Fig. 2) consisted of 75 infants (22w, *n* = 39; 23w, *n* = 36) with a birth weight of 499 ± 63 g, and 576 ± 102 g (Table 1), and a survival to discharge home of 41% and 64%, at 22 and 23 weeks, respectively.

**Table 1:** Characteristics of cohort with a gestational age of 22-23 weeks (*n* = 75).

Demography	Preeclampsia	3 (4)
	Premature rupture of membranes	29 (39)
	Antenatal steroids	
	Any	70 (93)
	Complete course	45 (60)
	Cesarean section	16 (21)
	22 weeks gestation	39 (52)
	Outborn	12 (16)
	Male	41 (55)
	Birth weight (g)	536 ± 92
	Apgar <4 at 10 min.	19 (25)
Outcomes	Intraventricular hemorrhage (grade III-IV)	16 (21)
	Necrotizing enterocolitis (surgical)	11 (16)
	Survival	39 (52)
	Postnatal age at death (days)	10 (4-22)
Endotracheal tube position*	Correct	54 (75)
	Distance to carina (mm)	7 (4-9)
	Corresponding vertebra	Th 2 (Th1-Th3)
	Incorrect	
	Deep	11 (15)
	High	7 (10)

Data presented as *n* (%), mean ± SD, or median (IQR); *n = 72.

### Endotracheal tube

The initial ETT positions are displayed in Table 1 and Supplement 1, and were correct in 67% of infants born at 22 weeks, and in 84% of infants born at 23 weeks. Table 2 displays the characteristics of infants with a too deeply positioned ETT versus the rest of the cohort. Although no statistically significant association was found between BW or GA and ETT position, the group with a too deeply positioned ETT had lower (*p* = 0.018) birth weight, and a majority (73%) were born at 22 weeks.

**Table 2:** Comparison of infants with correct vs too deep initial endotracheal tube position.

	Correct^†^ (*n* = 61)	Deep (*n* = 11)	*P*^
All infants (*n* = 72)			
Birth weight (g)	542 ± 93	485 ± 73	0.018
22 weeks’ gestation	31 (51)	8 (73)	ns
Apgar <4 at 10 min.	15 (24)	2 (18)	ns
Respiratory severity score*			
Day 1	2.4 (1.7–3.9)	2.6 (1.5–4.8)	ns
Day 7	4.4 (3.4–6.2)	4 (3.5–4.7)	ns
Age at first extubation (days)	3 (1–6)	4 (1–13)	ns
Survived	31 (51)	5 (45)	ns
Surviving infants (*n* = 39)			
Age at weaning (days)	47 (32–75)	55 (43–67)	ns
Mechanical ventilation (days)	38 (25–54)	38 (13–52)	ns
Bronchopulmonary dysplasia, grade 3	4 (5)	0	ns

Data presented as mean (SD), *n* (%), or median (IQR). ^†^Includes endotracheal tubes placed too high (*n* = 7). ^Mann-Whitney U-test. *Respiratory severity score = Mean airway pressure x Fraction of Inspired Oxygen.

Seven infants had a too high ETT position, of whom five were born at 22 weeks GA.

Chest and abdominal X-rays were taken at a median postnatal age of 117 (IQR 75–210) minutes.

### Umbilical artery catheter

Sixty-six infants had a UAC placed, with 21/66 (32%) being in a correct position. Of these, 11 were positioned high and required no further adjustment. According to local practice with use of a low UAC position, 10/55 (18%) were found to be correct, and the rest required adjustment after review of the X-rays (Supplement 2). The median measured span in distance for a correctly placed UAC was 13 (IQR 12–14) mm for a high position (at T6-T9), and 9 (IQR 8–10) mm for a low position (at L3-L4). The UACs were kept for a median of 5 (IQR 3–6) days. One UAC was placed too superficially.

### Umbilical vein catheter

Seventy infants had a UVC placed, with 2 (3%) being too superficial, 11 (16%) malpositioned in the liver, and 50 (71%) had a deep position (Supplement 3). The measured distance required for tip placement at the level of the diaphragm corresponded to the vertebral bodies of T8–T10, with a median insertion depth of 5.5 (IQR 5.0–6.1) cm from the abdominal skin surface. The UVCs were kept for a median of 6 (IQR 5–8) days. Ten infants died before UVC removal and in the remaining 59 cases, the UVC was replaced by a peripherally inserted central venous catheter.

The measured accurate insertion depths (Table 3) corresponded well with the standardized protocol for both ETT and UC placements, and all erroneously placed ETTs were due to deviations from the protocol.

**Table 3:** Measured accurate insertion depths in infants with a gestational age of 22-23 weeks.

		22 weeks	23 weeks
Endotracheal tube	Measured depth (cm)	5.5 (5.5)	5.8 (5.5-6.0)
	Corresponding vertebra	Th 2 (Th1-Th2)	Th 2 (Th1-Th2)
Umbilical artery catheter	Measured depth (cm)		
	Low position	5.8 (5.5–6.1)	6.5 (5.5–7)
	High position	9.5 (9.2–10.2)	10 (9.5–10.4)
Umbilical venous catheter	Measured depth (cm)	5.3 (5.0–5.8)	5.6 (5.2–6.2)

Data are Median (IQR). *Th* Thoracic.

### Respiratory outcomes

For the full cohort, the median age at first extubation was 2 (IQR 0–6), and the duration of mechanical ventilation 41 (IQR 28–54) days. Table 2 shows the outcomes of infants with a too deeply positioned ETT versus the rest of the cohort. No significant associations with ETT positioning were found in regression analysis adjusted for GA and BW (Table 4).

**Table 4:** Logistic and multivariable regression: too deep endotracheal tube positioning versus respiratory outcomes and death.

Outcome	Effect estimate	95% CI	*p*
All infants (*n* = 72)			
Respiratory severity score			
Day 1	β −0.03	−1.65; 1.60	0.975
Day 7	β −0.73	−2.75; 1.28	0.468
Age at first extubation (days)	β −6.86	−25.28; 11.55	0.456
Death	OR 0.97	0.25; 3.70	0.960
Surviving infants (*n* = 39)			
Age at weaning (days)	β −14.37	−47.20; 18.45	0.379
Mechanical ventilation (days)	β −5.51	−28.03; 17.01	0.627
Bronchopulmonary dysplasia, grade 3	NA*		

Adjusted for gestational age and birth weight. *No infant had BPD (grade 3).

## Discussion

This investigation presents a detailed radiological evaluation, exclusively in infants born at 22-23 weeks gestation, of standardized DR placement of endotracheal tubes and umbilical catheters. In our cohort of infants born at ≤23 weeks gestation, an initial ETT insertion depth of 5.5 cm at the lip was found to be accurate, and for umbilical lines the corresponding accurate depths of insertion were 6 cm (UAC, low position), and 5.5 cm (UVC) from the abdominal skin surface.

We found no significant associations between a too deep initial ETT position and respiratory outcomes or mortality. Nevertheless, in view of previously described risks with erroneously placed ETTs in more mature infants,^28–31^ we believe that a uniform application of the suggested insertion depths might contribute to an improved initial care of this vulnerable neonatal intensive care population.

The ideal method for estimating ETT insertion length in extremely preterm infants has been difficult to define. The non-linear relationship between optimal ETT length and weight, makes linear weight-based formulae, such as the 7-8-9 rule, unreliable,^25,28,30^ and several studies have found that such an approach overestimates ETT length in infants less than 750 g, and results in a high rate of deep positioning.^30,32^ The predecessor to the 7-8-9 rule, presented by Tochen et al. ^11^, studied 40 infants, with only 10 weighing less than 1000 g, and none weighing less than 700 g. Leung et al. ^33^ showed a linear relationship between ETT depth and both infant weight and length, but concluded length to be a reliable alternative during resuscitation, given the difficulty of quickly obtaining a reliable weight estimate.^34^ Numerous methods beyond weight and gestation have been proposed for predicting ETT length, including head and chest circumference,^33^ naso-tragal length,^19^ and foot length,^35^ but none have gained widespread clinical application.

Altogether, most previous data include cohorts consisting of considerably more mature/larger infants,^10,31^ limiting their relevance to infants born at less than 24 weeks, and also others have suggested the use of GA to determine insertion depth.^29^ Indeed, the application of the above weight (or length)-based formulae to our cohort would have resulted in a too deep ETT position in most, if not all, cases.

In our practice, umbilical catheters are routinely inserted deeper than recommended and adjusted after radiographic verification, as reflected by the need for repositioning in most cases. Consequently, we were unable to correlate the initial position of umbilical lines with BW or GA. However, the analysis of measured optimal insertion depth and BW revealed no linear relationship. On the other hand, our protocol, which is based on GA, aligned well with the accurate positions found in our analyses.

Umbilical catheter insertion depth based on anatomical measurements was first established by Dunn et al. in a postmortem study.^18^ Their findings, along with subsequent confirmatory studies, demonstrated the relationship between the shoulder-to-umbilicus distance and catheter length, forming the basis for widely used nomograms.^1,17^ As stated above regarding the ETT, compared to our cohort these studies included more mature infants. Shukla et al. ^19^ explored the relationship between BW and UC insertion depth, leading to a BW-based formula, which was later modified by Verheij et al. ^20^ and Wright et al. ^26^ for UVC and UAC, respectively. While these modifications better align with our findings, catheterization is often performed before birth weight is obtained.

A Cochrane review by Barrington et al. ^4^ reported fewer vascular complications and catheter removals with high UAC placement. While we could not analyze any such associations due to the small sample, it should be noted that a high position allows for slightly wider range of insertion depth, according to our measurements.

Our findings are in contrast to those of several previous studies that have reported an association between malpositioning of ETT, and adverse respiratory outcomes.^29,36^ Thayyil et al. found a significant increase in adverse pulmonary outcomes including pneumothorax, localized pulmonary interstitial emphysema, and right upper lobe and left lung collapse, in infants with a too deeply positioned ETT.^28^ Similarly, Pereira et al. demonstrated that infants with a too deeply positioned ETT required a longer duration of invasive ventilation.^30^ It should be noted that in the latter study, incorrect positioning was more frequent (> 50% of cases), compared to our data (15%). In our study radiographs were taken within 2 h of age, thus enabling a relatively brief period of suboptimal ventilation that might have minimized any negative effects of uneven ventilation/lung expansion. Further, while complications related to erroneous umbilical catheter positioning have been previously reported in 6–8%,^37^ we had no case of cardiac arrhythmia, tamponade, or hepatic necrosis.

One limitation with a retrospective study is that factors not accounted for might have impacted the data and their interpretation. One such is the head position during X-ray. Although the protocol recommends that X-rays should be taken with the head in midline neutral position, we cannot guarantee that this was the actual case in all instances. Although the optimal ETT tip position has been suggested to be mid-tracheal, between T1 to T2,^25,38^ radiological landmarks may vary^7^ particularly in the tiniest infants. Indeed, since the distance from carina to T1 can be less than 10 mm in this population,^7^ even small differences in head/neck position could impact ETT tip position. Another limitation is the documentation of ETT/UC position where we cannot rule out that it could have been erroneously reported in some cases. The small sample is another obvious limitation that precludes any definitive conclusions about the potential effects of suboptimal ventilation in relation to a too deep ETT position.

Real-time guidance via ultrasound have been shown to be superior to chest X-ray for verifying UC tip position and minimizing adverse outcomes,^39–41^ and could have improved catheter positioning in our study. However, few infants under 750 g were included in those studies,^39–41^ and this approach is presently not used in our unit during DR management.

## Conclusions

The findings of this retrospective cohort study of infants born at the lowest extremely preterm infant spectrum, demonstrate that the use of standardized gestational age-based insertion depths for ETT and umbilical lines is feasible. The suggested insertion depths can be expected to result in an accurate ETT position in infants born at 22-23 weeks of gestation, regardless of birth weight.

## Supplementary information

Supplemental material

## Data Availability

All relevant data for this study are included in the article. Further enquiries can be directed to the corresponding author.
