# Supplementary material for: Standardized endotracheal tube and intravascular access placement in infants born at 22-23 weeks gestation
Source: Pediatr Res. 2025 Jun 18;99(5):1836–41. doi: 10.1038/s41390-025-04186-8 (PMC13221281; doi:10.1038/s41390-025-04186-8)
Supplement: Supplementary file 1 — Supplemental material [file 41390_2025_4186_MOESM1_ESM.pdf]

Delivery Room Management Protocol

Antepartum

Mothers approaching 22 weeks with threatening labor are referred for obstetric care with the intention to prolong the pregnancy, and allow for the effect of antenatal steroid treatment, which can be administered at 21+6 weeks. If labor is imminent and prenatal transport cannot be safely conducted, a dedicated transport team (neonatologist/neonatal nurse) is dispatched from the regional center to retrieve the infant from any of the eight other delivery hospitals in the region. According to the guidelines from the Swedish Society of Obstetrics and Gynecology and the Swedish Neonatal Society, cesarean section is not performed for fetal indication prior to 23 weeks.

The standardized **DR management protocol** at 22-23 weeks gestation is as follows:

a) Staff (attending neonatologist, neonatal nurse and assistant nurse).
b) Immediate DR intubation (oral route) with a size 2.0F endotracheal tube (ETT) at a lip-tip distance of 5.5 cm
c) All respiratory support provided with a neonatal ventilator (Sophie®, Fritz Stephan GmbH, Gackenbach, Germany), with default settings (Synchronized intermittent mandatory ventilation; a peak inspiratory pressure of 20 cm H_2O, a positive end-expiratory pressure of 4 cm H_2O, an inspiratory: expiratory ratio of 1:2, and a respiratory rate of 60/min)
d) Early instillation of 1ml Poractant alfa in the DR (Curosurf® 80 mg/ml, Chiesi Farmaceutici, Parma, Italy)
e) After achieving adequate chest rise, heart rate, and oxygen saturation, settings are switched to volume-targeted ventilation with an initial target tidal volume of 2.5 ml (5 ml/kg)
f) Placement of umbilical catheters (arterial and venous) to a depth of 6 cm from the skin surface
g) Transfer of the infant to the NICU with unchanged ventilator settings.
h) Chest and abdominal X-rays promptly performed at NICU admission to verify the positions of the ETT and umbilical catheters.

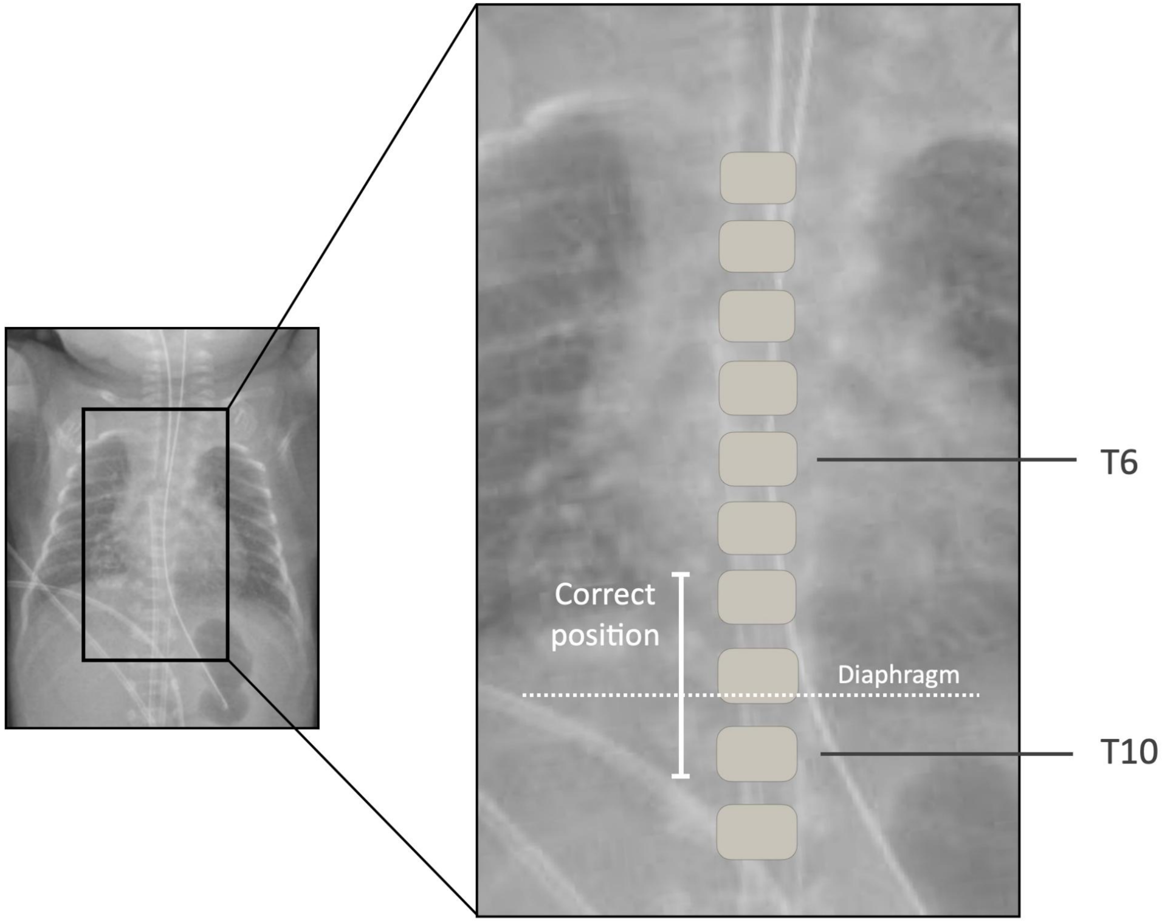

Figure S1. Representative chest x-ray with enlarged box to illustrate radiological features of umbilical venous catheter (UVC) position (T, thoracic).

Supplement 1

Endotracheal tube positioning in infants with a gestational age of 22-23 weeks

	22 w (n=39)	23 w (n=33)
Correct position	26 (67)	28 (84)
Distance to carina (mm)	6 (4-8)	7 (4-9)
Corresponding vertebra	Th 2 (Th1-Th3)	Th 2 (Th1-Th3)
Incorrect position		
Too deep	8 (21)	3 (8)
Too short	5 (13)	2 (6)

Data are n (%), or Median (IQR).

Supplement 2

Umbilical artery catheter positioning in infants with a gestational age of 22-23 weeks (n=66)

	22-23 w	22 w	23 w
Low position	n=55	n=28	n=27
Correct	10 (18)	6 (21)	4 (15)
Incorrect	45 (82)	22 (79)	23 (85)
High position	n=11	n=8	n=3
Correct	11 (100)	8 (100)	3 (100)
Incorrect	0		

Data are n (%)

Supplement 3

Umbilical venous catheter positioning in infants with a gestational age of 22-23 weeks

	22-23 w (n=70)	22 w (n=34)	23 w (n=36)
Correct position	7 (10)	4 (12)	3 (8)
Incorrect position	52 (74)	26 (77)	26 (72)
Malposition (in liver)	11 (16)	4 (12)	7 (19)

Data are n (%)
